# Poly[(μ_4_-decanedio­ato)cobalt(II)]

**DOI:** 10.1107/S1600536814006011

**Published:** 2014-04-02

**Authors:** Bruno Giuseppe, Nicolò Francesco, Grassi Giovanni, Saccà Alessandro, Viviana Mollica Nardo

**Affiliations:** a Dipartimento di Scienze Chimiche, Universita di Messina, Messina, Italy

## Abstract

In the title compound, [Co(C_10_H_16_O_4_)]_*n*_, the Co^II^ atom is bonded in a slightly distorted tetra­hedral environment by four O atoms from the bridging sebacate dications, comprising two separate half-ligands which lie across crystallographic inversion centres. In the three-dimensional network coordination polymer, there are two different spatial extensions of Co^II^ atoms, one with the Co^II^ atoms lying parallel to (100) [Co⋯Co = 4.653 (1) Å], the other lying parallel to (010) [Co⋯Co = 4.764 (1) Å].

## Related literature   

For background to the construction of supra­molecular frameworks, see: Gavezzotti (1994 ▶); Desiraju (2003 ▶); Sarma & Desiraju (2002 ▶); Biradha *et al.* (1998 ▶); Hosseini (2003 ▶). For the structure of sebacic acid, see: Morrison & Robertson (1949 ▶); Bond *et al.* (2001 ▶). For its use in constructing stable metal-organic frameworks, see: Borkowski & Cahill (2004 ▶, 2006 ▶); Thuéry (2008 ▶); Zhou *et al.* (2010 ▶).
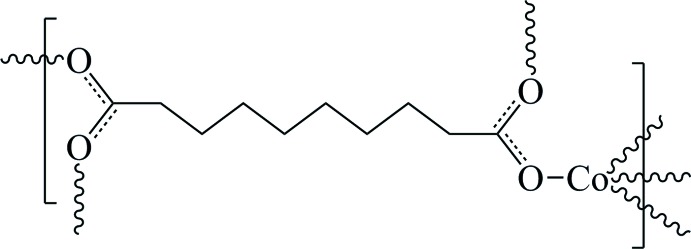


## Experimental   

### 

#### Crystal data  

[Co(C_10_H_16_O_4_)]
*M* *_r_* = 259.16
Monoclinic, 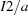

*a* = 9.276 (1) Å
*b* = 4.764 (1) Å
*c* = 50.154 (3) Å
β = 95.02 (2)°
*V* = 2207.9 (5) Å^3^
*Z* = 8
Mo *K*α radiation
μ = 1.55 mm^−1^
*T* = 295 K
0.28 × 0.21 × 0.17 mm

#### Data collection  

Bruker APEXII CCD diffractometer
Absorption correction: multi-scan (*SADABS*; Sheldrick, 2004 ▶) *T*_min_ = 0.661, *T*_max_ = 0.746
32758 measured reflections
2477 independent reflections
2175 reflections with *I* > 2σ(*I*)
*R*_int_ = 0.044

#### Refinement  

*R*[*F* ^2^ > 2σ(*F* ^2^)] = 0.047
*wR*(*F* ^2^) = 0.157
*S* = 1.01
2477 reflections
136 parameters
H-atom parameters constrained
Δρ_max_ = 0.59 e Å^−3^
Δρ_min_ = −1.13 e Å^−3^

### 

Data collection: *APEX2* (Bruker, 2009 ▶); cell refinement: *SAINT* (Bruker, 2009 ▶); data reduction: *SAINT*; program(s) used to solve structure: *SHELXS97* (Sheldrick, 2008 ▶); program(s) used to refine structure: *SHELXL97* (Sheldrick, 2008 ▶); molecular graphics: *SHELXTL* (Sheldrick, 2008 ▶); software used to prepare material for publication: *SHELXTL*.

## Supplementary Material

Crystal structure: contains datablock(s) I, New_Global_Publ_Block. DOI: 10.1107/S1600536814006011/zs2284sup1.cif

Structure factors: contains datablock(s) I. DOI: 10.1107/S1600536814006011/zs2284Isup2.hkl

CCDC reference: 992380

Additional supporting information:  crystallographic information; 3D view; checkCIF report

## Figures and Tables

**Table 1:** Selected bond lengths (Å)

Co1—O1	1.968 (3)
Co1—O2	1.953 (3)
Co1—O3^i^	1.972 (3)
Co1—O4^ii^	1.963 (3)

Symmetry codes: (i) 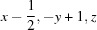; (ii) 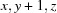.

**Fig. 1.**
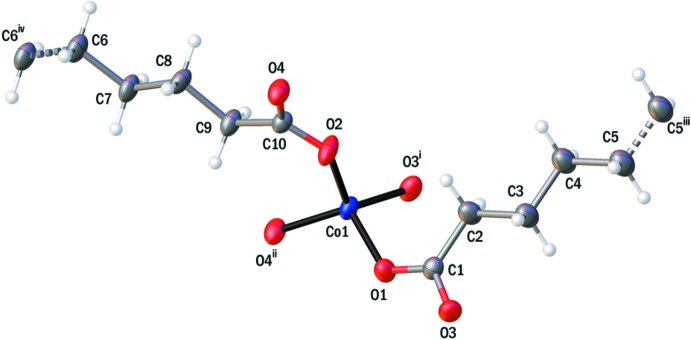
Molecular configuration and atom numbering for the title complex with non H-atoms represented as displacement ellipsoids plotted at the 50% probability level and H atoms shown as small spheres of arbitrary radius. The broken bonds C5—C5iii and C6—C6iv link the inversion-related halves of the sebacate ligands. For symmetry codes (i) and (ii), see Table 1. For other codes: (iii) -x + 3/2, -y - 1/2, -z + 1/2; (iv) -x, -y, -z.

**Fig. 2.**
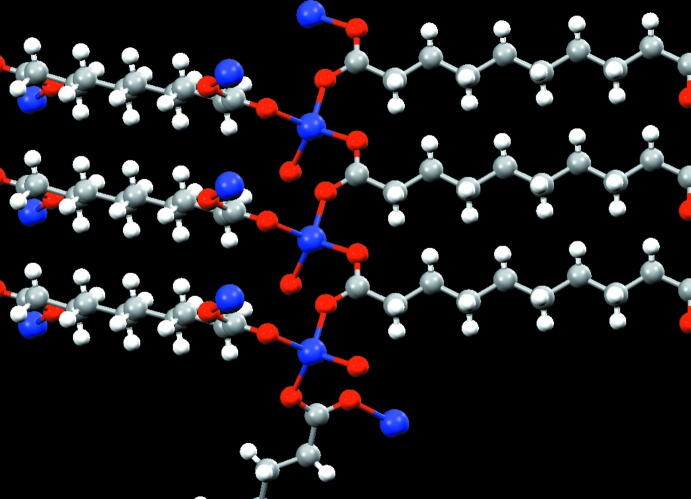
Perspective view of the three-dimensional network structure, showing the polymeric extensions.

**Fig. 3.**
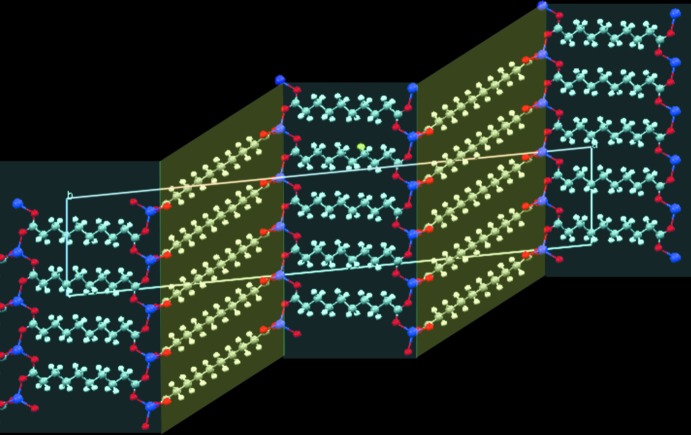
Packing diagram of the three-dimensional compound viewed along the b axis.

## supplementary crystallographic information

### 1. Comment

Crystal engineering is primarily concerned with the ability to predictably synthesize supramolecular structures from well designed building-blocks (Desiraju, 2003). To date it is still a big challenge the exact prediction of the structure of a molecular solid because crystal packing is driven by many weak non-covalent interactions (Gavezzotti, 1994). Suitable substrates to design specific architectures should bear functional groups apt to develop predefined interactions synthones (Sarma & Desiraju, 2002) for this purpose, often, planar aromatic or linear aliphatic molecules with carboxylic groups (Biradha *et al.*, 1998) were exploited as building-blocks (Hosseini, 2003) to yield particular crystal lattice. Crystal structure of sebacic acid was first determined by Morrison & Robertson (1949) it has been redetermined at low temperature (180 K) (Bond *et al.*, 2001). Sebacic acid both in its protonated or deprotonated forms has been found in several metal complexes, either coordinated to the metal center or as a counter ion, or in co-crystals in its protonated form. In all the examined compounds, the alkyl chain of either the free or coordinated sebacate or sebacic acid are usually linear with a few exceptions (Zhou *et al.*, 2010; Thuéry, 2008). In the title zinc sebacate complex, [CoC_10_H_16_O_4_]_n_ the linear chain is evidenced by the C1–C10 separation of 11.452 (4) Å, equal within the e.s.d's to the corresponding value of 11.466 (5) Å found in the low- temperature X-ray structure of sebacic acid (Bond *et al.*, 2001).The shortest separation [11.419 (4) Å] for the linear C1···C10 chain was found in a dimeric uranil sebacate complex (Borkowski & Cahill, 2006).

The asymmetric unit of the title complex comprises a cobalt cation coordinated by four carboxyl O-atom donors from two non-equivalent half-sebacate anions which lie across crystallographic inversion centres (Fig. 1). The cobalt has close to ideal tetrahedral geometry [Co—O range, 1.953 (3) – 1.972 (3) Å (Table 1)]. The C—O bond lengths in the carboxylate groups range from 1.252 (5) Å to 1.262 (5) Å, this narrow range being smaller than the usual range found in monodentate carboxylates. The title complex forms a three-dimensional network polymer in which there are two different arrangenments of cobalt atoms (Fig. 2). The column of cobalt atoms with the oxygen atoms linked to it extends parallel to the crystallographic *b* axis and in this column the Co–Co separation is exactly the length of *b* axis [4.7640 (7) Å]. The second column extends almost parallel to (1 0 0) with a Co···Co separation of 4.6528 (8) Å. The overall molecular packing is illustrated in Fig. 3.

### 2. Experimental

The polymer was synthesized by reaction of cobalt chloride hexahydrate (0.05 mmol) with sebacic acid (0.05 mmol) sealed in a teflon-lined stainless steel autoclave filled with 8 ml of water, which was heated at 130 °C for 3 days under autogenous pressure. After slow cooling to room temperature over 6 h, two different types of crystal were observed, the expected pink violet product, the title complex (yield 50%) and transparent colourless crystals, which tested separately appear to be unreacted sebacic acid.

### 3. Refinement

The H atoms were included in the refinement at calculated positions [C—H = 0.97 Å] and were allowed to ride, with *U*_eq_(H) = 1.2*U*_eq_(H).

### Crystal data

**Table:**

[Co(C_10_H_16_O_4_)]	*F*(000) = 1080
*M**_r_* = 259.16	*D*_x_ = 1.559 Mg m^−^^3^
Monoclinic, *I*2/*a*	Mo *K*α radiation, λ = 0.71073 Å
Hall symbol: -I 2ya	Cell parameters from 98 reflections
*a* = 9.276 (1) Å	θ = 2.2–27.5°
*b* = 4.764 (1) Å	µ = 1.55 mm^−^^1^
*c* = 50.154 (3) Å	*T* = 295 K
β = 95.02 (2)°	Prismatic, pink
*V* = 2207.9 (5) Å^3^	0.28 × 0.21 × 0.17 mm
*Z* = 8	

### Data collection

**Table:**

Bruker APEXII CCD diffractometer	2477 independent reflections
Radiation source: fine-focus sealed tube	2175 reflections with *I* > 2σ(*I*)
Graphite monochromator	*R*_int_ = 0.044
φ and ω scans	θ_max_ = 27.5°, θ_min_ = 0.8°
Absorption correction: multi-scan (*SADABS*; Sheldrick, 2004)	*h* = −12→12
*T*_min_ = 0.661, *T*_max_ = 0.746	*k* = −6→6
32758 measured reflections	*l* = −65→65

### Refinement

**Table:**

Refinement on *F*^2^	Primary atom site location: structure-invariant direct methods
Least-squares matrix: full	Secondary atom site location: difference Fourier map
*R*[*F*^2^ > 2σ(*F*^2^)] = 0.047	Hydrogen site location: inferred from neighbouring sites
*wR*(*F*^2^) = 0.157	H-atom parameters constrained
*S* = 1.01	*w* = 1/[σ^2^(*F*_o_^2^) + (0.0975*P*)^2^ + 11.5316*P*] where *P* = (*F*_o_^2^ + 2*F*_c_^2^)/3
2477 reflections	(Δ/σ)_max_ = 0.005
136 parameters	Δρ_max_ = 0.59 e Å^−^^3^
0 restraints	Δρ_min_ = −1.13 e Å^−^^3^

### Special details

**Table:**

Geometry. Bond distances, angles etc. have been calculated using the rounded fractional coordinates. All su's are estimated from the variances of the (full) variance-covariance matrix. The cell esds are taken into account in the estimation of distances, angles and torsion angles.
Refinement. Refinement of *F*^2^ against ALL reflections. The weighted *R*-factor *wR* and goodness of fit *S* are based on *F*^2^, conventional *R*-factors *R* are based on *F*, with *F* set to zero for negative *F*^2^. The threshold expression of *F*^2^ > 2σ(*F*^2^) is used only for calculating *R*-factors(gt) *etc*. and is not relevant to the choice of reflections for refinement. *R*-factors based on *F*^2^ are statistically about twice as large as those based on *F*, and *R*- factors based on ALL data will be even larger. Structure has been solved and refined in the centrosymmetric monoclinic *C*2/*c* space group. Refining the structure in the non standard I2/a space group leads to identical *R* value results, but at a value of G.o.f. (1.014) significantly closer to the ideal value of 1, for this reason we prefer the non-standard space group.

### Fractional atomic coordinates and isotropic or equivalent isotropic displacement parameters (Å^2^)

**Table:**

	*x*	*y*	*z*	*U*_iso_*/*U*_eq_	
Co1	0.47978 (6)	0.46111 (10)	0.12353 (1)	0.0250 (2)	
O1	0.6900 (3)	0.4833 (6)	0.13284 (6)	0.0309 (8)	
O2	0.4577 (4)	0.0653 (6)	0.11391 (6)	0.0366 (9)	
O3	0.8963 (3)	0.3988 (7)	0.15584 (6)	0.0323 (8)	
O4	0.4116 (3)	−0.3222 (6)	0.09159 (6)	0.0325 (8)	
C1	0.7614 (4)	0.3735 (8)	0.15271 (7)	0.0257 (10)	
C2	0.6850 (4)	0.2035 (10)	0.17252 (8)	0.0354 (11)	
C3	0.7826 (5)	0.0818 (10)	0.19561 (9)	0.0376 (14)	
C4	0.6969 (5)	−0.0629 (11)	0.21590 (10)	0.0405 (14)	
C5	0.7919 (5)	−0.1789 (11)	0.23957 (9)	0.0418 (14)	
C6	0.0421 (5)	−0.0875 (10)	0.01058 (9)	0.0363 (12)	
C7	0.1253 (5)	0.0870 (9)	0.03230 (8)	0.0354 (12)	
C8	0.2129 (4)	−0.0888 (9)	0.05305 (8)	0.0331 (11)	
C9	0.2964 (5)	0.0920 (9)	0.07393 (8)	0.0323 (12)	
C10	0.3946 (4)	−0.0612 (7)	0.09434 (8)	0.0246 (10)	
H2A	0.63420	0.05040	0.16300	0.0430*	
H2B	0.61290	0.32160	0.17980	0.0430*	
H3A	0.84870	−0.05180	0.18860	0.0450*	
H3B	0.83970	0.23150	0.20440	0.0450*	
H4A	0.64190	−0.21550	0.20720	0.0490*	
H4B	0.62870	0.06970	0.22240	0.0490*	
H5A	0.85920	−0.31300	0.23300	0.0510*	
H5B	0.84820	−0.02640	0.24800	0.0510*	
H6A	0.10990	−0.20510	0.00200	0.0440*	
H6B	−0.02450	−0.21000	0.01890	0.0440*	
H7A	0.19000	0.21330	0.02400	0.0420*	
H7B	0.05720	0.20070	0.04120	0.0420*	
H8A	0.28010	−0.20510	0.04420	0.0400*	
H8B	0.14830	−0.21190	0.06180	0.0400*	
H9A	0.35410	0.22490	0.06480	0.0390*	
H9B	0.22750	0.19920	0.08330	0.0390*	

### Atomic displacement parameters (Å^2^)

**Table:**

	*U*^11^	*U*^22^	*U*^33^	*U*^12^	*U*^13^	*U*^23^
Co1	0.0240 (3)	0.0266 (3)	0.0228 (3)	0.0029 (2)	−0.0062 (2)	−0.0017 (2)
O1	0.0254 (14)	0.0385 (15)	0.0282 (14)	0.0012 (10)	−0.0013 (11)	0.0082 (11)
O2	0.0502 (19)	0.0249 (14)	0.0306 (15)	0.0056 (12)	−0.0197 (13)	−0.0032 (11)
O3	0.0215 (13)	0.0426 (16)	0.0320 (14)	0.0037 (11)	−0.0013 (10)	−0.0053 (12)
O4	0.0408 (15)	0.0236 (14)	0.0309 (14)	0.0030 (11)	−0.0095 (11)	0.0003 (10)
C1	0.0223 (16)	0.0287 (18)	0.0258 (17)	0.0032 (14)	−0.0001 (13)	−0.0015 (14)
C2	0.0247 (18)	0.047 (2)	0.034 (2)	−0.0021 (16)	0.0002 (15)	0.0122 (17)
C3	0.027 (2)	0.051 (3)	0.034 (2)	−0.0023 (17)	−0.0013 (16)	0.0137 (18)
C4	0.030 (2)	0.056 (3)	0.035 (2)	−0.0023 (18)	0.0000 (17)	0.0147 (19)
C5	0.033 (2)	0.055 (3)	0.037 (2)	−0.0004 (19)	0.0009 (17)	0.015 (2)
C6	0.031 (2)	0.042 (2)	0.033 (2)	0.0033 (17)	−0.0143 (17)	0.0003 (17)
C7	0.032 (2)	0.040 (2)	0.031 (2)	0.0028 (17)	−0.0148 (16)	0.0004 (16)
C8	0.0292 (19)	0.033 (2)	0.034 (2)	−0.0009 (15)	−0.0148 (16)	−0.0002 (16)
C9	0.034 (2)	0.030 (2)	0.030 (2)	0.0037 (16)	−0.0135 (15)	−0.0003 (15)
C10	0.0206 (17)	0.0259 (18)	0.0263 (18)	0.0021 (13)	−0.0037 (14)	−0.0008 (13)

### Geometric parameters (Å, º)

**Table:**

Co1—O1	1.968 (3)	C9—C10	1.499 (6)
Co1—O2	1.953 (3)	C2—H2A	0.9700
Co1—O3^i^	1.972 (3)	C2—H2B	0.9700
Co1—O4^ii^	1.963 (3)	C3—H3A	0.9700
O1—C1	1.261 (5)	C3—H3B	0.9700
O2—C10	1.252 (5)	C4—H4A	0.9700
O3—C1	1.253 (5)	C4—H4B	0.9700
O4—C10	1.262 (4)	C5—H5A	0.9700
C1—C2	1.506 (6)	C5—H5B	0.9700
C2—C3	1.521 (6)	C6—H6A	0.9700
C3—C4	1.511 (7)	C6—H6B	0.9700
C4—C5	1.519 (7)	C7—H7A	0.9700
C5—C5^iii^	1.517 (7)	C7—H7B	0.9700
C6—C7	1.525 (6)	C8—H8A	0.9700
C6—C6^iv^	1.512 (7)	C8—H8B	0.9700
C7—C8	1.516 (6)	C9—H9A	0.9700
C8—C9	1.515 (6)	C9—H9B	0.9700
			
O1—Co1—O2	101.00 (14)	C4—C3—H3B	109.00
O1—Co1—O4^ii^	114.02 (12)	H3A—C3—H3B	108.00
O1—Co1—O3^i^	103.83 (12)	C3—C4—H4A	109.00
O2—Co1—O4^ii^	106.66 (13)	C3—C4—H4B	109.00
O2—Co1—O3^i^	119.31 (14)	C5—C4—H4A	109.00
O3^i^—Co1—O4^ii^	111.77 (13)	C5—C4—H4B	109.00
Co1—O1—C1	127.3 (3)	H4A—C4—H4B	108.00
Co1—O2—C10	133.6 (3)	C4—C5—H5A	109.00
Co1^v^—O3—C1	112.9 (2)	C4—C5—H5B	109.00
Co1^vi^—O4—C10	117.6 (3)	H5A—C5—H5B	108.00
O1—C1—O3	120.6 (3)	C5^iii^—C5—H5A	109.00
O1—C1—C2	120.0 (3)	C5^iii^—C5—H5B	109.00
O3—C1—C2	119.4 (3)	C7—C6—H6A	109.00
C1—C2—C3	115.1 (3)	C7—C6—H6B	109.00
C2—C3—C4	111.9 (4)	H6A—C6—H6B	108.00
C3—C4—C5	112.9 (4)	C6^iv^—C6—H6A	109.00
C4—C5—C5^iii^	113.8 (4)	C6^iv^—C6—H6B	109.00
C6^iv^—C6—C7	113.5 (4)	C6—C7—H7A	109.00
C6—C7—C8	113.4 (4)	C6—C7—H7B	109.00
C7—C8—C9	111.8 (4)	C8—C7—H7A	109.00
C8—C9—C10	116.0 (3)	C8—C7—H7B	109.00
O2—C10—O4	120.4 (4)	H7A—C7—H7B	108.00
O2—C10—C9	121.0 (3)	C7—C8—H8A	109.00
O4—C10—C9	118.6 (3)	C7—C8—H8B	109.00
C1—C2—H2A	108.00	C9—C8—H8A	109.00
C1—C2—H2B	108.00	C9—C8—H8B	109.00
C3—C2—H2A	109.00	H8A—C8—H8B	108.00
C3—C2—H2B	109.00	C8—C9—H9A	108.00
H2A—C2—H2B	107.00	C8—C9—H9B	108.00
C2—C3—H3A	109.00	C10—C9—H9A	108.00
C2—C3—H3B	109.00	C10—C9—H9B	108.00
C4—C3—H3A	109.00	H9A—C9—H9B	107.00
			
O2—Co1—O1—C1	−68.6 (3)	Co1^v^—O3—C1—C2	164.7 (3)
O4^ii^—Co1—O1—C1	177.4 (3)	Co1^vi^—O4—C10—O2	20.7 (5)
O3^i^—Co1—O1—C1	55.5 (3)	Co1^vi^—O4—C10—C9	−158.6 (3)
O1—Co1—O2—C10	−130.9 (4)	O1—C1—C2—C3	180.0 (4)
O4^ii^—Co1—O2—C10	−11.5 (4)	O3—C1—C2—C3	1.7 (6)
O3^i^—Co1—O2—C10	116.2 (4)	C1—C2—C3—C4	174.8 (4)
O1—Co1—O4^ii^—C10^ii^	−80.3 (3)	C2—C3—C4—C5	−178.3 (4)
O2—Co1—O4^ii^—C10^ii^	169.1 (3)	C3—C4—C5—C5^iii^	179.1 (4)
O1—Co1—O3^i^—C1^i^	173.9 (3)	C4—C5—C5^iii^—C4^iii^	180.0 (4)
O2—Co1—O3^i^—C1^i^	−74.8 (3)	C6^iv^—C6—C7—C8	−178.4 (4)
Co1—O1—C1—O3	178.1 (3)	C7—C6—C6^iv^—C7^iv^	180.0 (4)
Co1—O1—C1—C2	−0.2 (5)	C6—C7—C8—C9	178.8 (4)
Co1—O2—C10—O4	171.7 (3)	C7—C8—C9—C10	−176.0 (3)
Co1—O2—C10—C9	−9.0 (6)	C8—C9—C10—O2	−173.1 (4)
Co1^v^—O3—C1—O1	−13.6 (5)	C8—C9—C10—O4	6.2 (5)

Symmetry codes: (i) *x*−1/2, −*y*+1, *z*; (ii) *x*, *y*+1, *z*; (iii) −*x*+3/2, −*y*−1/2, −*z*+1/2; (iv) −*x*, −*y*, −*z*; (v) *x*+1/2, −*y*+1, *z*; (vi) *x*, *y*−1, *z*.
